# Radiologic criteria of retropharyngeal lymph node metastasis in maxillary sinus cancer

**DOI:** 10.1186/s13014-021-01917-z

**Published:** 2021-09-26

**Authors:** Yasuo Kosugi, Michimasa Suzuki, Mitsuhisa Fujimaki, Shinichi Ohba, Fumihiko Matsumoto, Yoichi Muramoto, Terufumi Kawamoto, Masaki Oshima, Naoto Shikama, Keisuke Sasai

**Affiliations:** 1 Department of Radiation Oncology, Juntendo University, 2-1-1 Hongo, Bunkyo-ku, Tokyo, 113-8421 Japan; 2 Department of Otorhinolaryngology, Head and Neck Surgery, Juntendo University, Tokyo, Japan; 3 Department of Radiology, Juntendo University, Tokyo, Japan

**Keywords:** Maxillary sinus cancer, Retropharyngeal lymph node metastasis, Radiologic criteria, Magnetic resonance image

## Abstract

**Objective:**

To determine the most appropriate radiologic criteria of metastatic retropharyngeal lymph nodes (RLNs) in patients with maxillary sinus cancer (MSC).

**Materials and methods:**

We retrospectively evaluated 16 consecutive patients who underwent magnetic resonance imaging (MRI) before and after the treatment of locally advanced squamous cell carcinoma of the maxillary sinus. The minimal and maximal diameters of all RLNS were recorded. RLNs were classified as metastatic on the basis of the MRI follow-up (f/u). RLNs were considered non-metastatic if stable disease continued until the final MRI f/u and metastatic in cases with different evaluations (complete response, partial response, progressive disease) determined using Response Evaluation Criteria in Solid Tumours (RECIST) ver. 1.1. The receiver operating characteristic curve (ROC) and area under the curve (AUC) were used to assess the accuracy of various criteria in the diagnosis of metastatic RLNs.

**Results:**

Of the 34 RLNs in 16 cases observed on pretreatment MRI, 7 were classified as metastatic RLNs and 27 as non-metastatic RLNs. Using the radiologic criteria, metastatic RLNs tended to be diagnosed more accurately with the minimal axial diameter than with the maximal axial diameter (AUC; 0.97 vs. 0.73, *p* = 0.06). The most accurate size criterion of metastatic RLNs was a minimal axial diameter of 5 mm or larger, with an accuracy of 94.1% (32 of 34).

**Conclusions:**

The most appropriate radiologic criterion of metastatic RLNs in MSC is a minimal axial diameter of 5 mm or longer.

## Introduction

Maxillary sinus cancer (MSC) is a relatively rare disease, with an incidence of 1 per 100 000 person-years, and accounts for 3% of all head and neck cancers. Most MSC cases are squamous cell carcinoma (SCC), representing 60% to 90% of total cases [1]. Tobacco use is a significant risk factor. The prognosis of MSC with lymph node (LN) metastases is significantly poor. Five-year overall survival (OS) of patients with SCC of the paranasal sinuses is approximately 50%, and 30% among those with localized and regional disease [2].

Surgery and (chemo)radiation therapy are recommended as the standard treatments for MCS [2]. The combination of radiation therapy and intra-arterial (IA) chemotherapy is a promising treatment for patients who had unresectableMSC or refused surgery because of its high control rate and high OS [3, 4]. This is a popular treatment in Japan, and although it is not a standard method, we have observed good results in patients treated with locally advanced MSC using this combination [5]. In this setting, we have performed elective nodal irradiation (ENI) only for patients with regional LN metastasis (cN +). From the results of a meta-analysis, the guidelines of the National Comprehensive Cancer Network (NCCN) recommended ENI for patients with advanced MSC [6], but ENI is controversial because of reports of increased late adverse events comprising dysphagia and trismus [7]. We reported that ENI increased the radiation dose in the pharyngeal contractile muscles and increased late aspiration pneumonia [8]. The irradiation field setting of the retropharyngeal lymph node (RLN) region is important because it affects the radiation dose of the pharyngeal contractile muscle and may cause severe acute mucositis and late dysphagia in ENI [9–12]. Recently, intensity-modulated radiation therapy (IMRT) for MSC has been reported to be useful in reducing adverse events [2]. Similarly, IMRT highlights the risk of marginal recurrence, and a more accurate irradiation field setting is required [13]. Because of the high frequency of metastatic RLNs detected by magnetic resonance imaging (MRI) in recent years [14], the European Society for Radiotherapy & Oncology (ESTRO) guidelines recommended including the RLN area as a prophylactic region for radiation therapy in MSC [15]. However, radiologic criteria for metastatic RLNs in MSC have not been established.

Therefore, in this study, we determined metastatic RLNs on the basis of the therapeutic response of RLNs using MRI images taken before and after treatment and investigated the appropriate radiologic criteria for metastatic RLNs in MSC.

## Patients and methods

### Study design and data collection

We retrospectively analyzed the clinical data of 16 consecutive patients who underwent MRI before and after treatment among 55 patients with locally advanced SCC of the maxillary sinus who were treated with definitive external beam radiotherapy and IA chemotherapy from April 2009 to August 2017 at our institution. All patients had unresectable MSC or refused surgery. The ethics committee of our hospital approved the study protocol (approval number: 19–173), and the study was conducted in accordance with the principles of the Declaration of Helsinki. Furthermore, staging was performed on the basis of findings from physical examinations, computed tomography (CT), MRI, and/or positron emission tomography-CT (PET-CT), in accordance with the Union for International Cancer Control (UICC, 7^th^ edition) [16].

### Treatment

Details of definitive external radiotherapy and super-selective IA chemotherapy were given in our previous report [7]. In cN + cases, the primary sites and metastatic LNs were irradiated with 60–70 Gy (median, 60 Gy, to iso-center by three-dimensional conformal radiation therapy (3DCRT), prescription dose > 50% of the target volume by IMRT) in 30–35 fractions over 6–7 weeks, and the prophylactic regional area (ENI) was irradiated with 46 Gy in 23 fractions by 3DCRT or 50 Gy in 25 fractions by IMRT (median, 46 Gy). ENI was not performed in clinical node-negative cases. Ipsilateral levels I, II, and III were set for regional irradiation, and contralateral levels II and III were also added in cases of bilateral LN metastases at presentation. Bilateral RLN regions were added for regional irradiation in IMRT cases.

### Imaging protocol

Of 16 patients, 11 underwent MR imaging with a 3-Tesla system (Achieva or Ingenia; Royal Philips, Amsterdam, Netherlands) employing a three-dimensional gradient-echo technique. The entire RLN region was examined with a head and neck-combined coil. After intravenous injection of gadolinium contrast agent (Magnescope; Guerbet Japan, Tokyo, Japan) at a dose of 0.2 mmol per kilogram of body weight, T1-weighted fat-suppressed axial, coronal, and sagittal sequences were performed sequentially. The most frequently used section thicknesses and intersection gaps were 2 mm and 0.9 mm, respectively, for the axial plane.

### Image assessment

All MRI images were evaluated by a radiation oncologist and a radiologist (Y.K. with 10 years of experience in MSC MRI imaging and radiation therapy, and M.S. with 19 years of experience in MSC MRI imaging and super-selective IA treatment). If there was a discrepancy between the findings, they were discussed, and a consensus result was recorded. Details of cranial level and ipsilateral or contralateral side were recorded for all visible RLNs at pre-treatment MRI. The minimal and maximal axial diameters of each RLN were measured. The minimal diameter was defined as the widest diameter of the LN in the axial plane that was perpendicular to the maximal axial diameter. In addition, findings of central necrosis (marginal ring-shaped contrast enhancement) of RLNs were also recorded as indicative of metastatic RLNs.

### Therapeutic response detected by MRI follow-up

The therapeutic response was first investigated by MRI in all patients within 8 to 12 weeks after the completion of radiation therapy. Then, MRI follow up (f/u) was undertaken within 3 to 12 months. Therapeutic response was classified into complete response (CR), partial response (PR), stable disease (SD), and progressive disease (PD) with reference to the Response Evaluation Criteria in Solid Tumors (RECIST) ver. 1.1, as follows: disappearance, CR; reduction in maximal axial diameter by 30% or more, PR; maximal axial diameter increased by 20% or more PD; no change, SD [17]. An RLN was considered as metastatic if it was classified as CR, PR, or PD at any MRI f/u, and was not considered a metastatic LN if SD continued until the final MRI f/u without local recurrence. For RLNs that were changed at the initial MRI after treatment but were evaluated as SD, when the reduction or increase continued in the subsequent additional MRI f/u, the pretreatment MRI and the most changed MRI image were compared.

### Statistical analysis

Receiver operating characteristic (ROC) curve and the area under the curve (AUC) were used to determine the most appropriate radiologic criterion of metastatic RLNs in MSC. The sensitivity, specificity, and accuracy were calculated using the standard definitions. The DeLong test was performed to compare the AUC of ROC curves. The most appropriate radiologic criterion was determined using the Youden’s index in conjunction with the ROC curve. The Mann–Whitney U test was used to compare the diameters of the metastatic or non-metastatic RLNs. All statistical analyses were assessed at a significance level of 0.05 using JMP 12 software (SAS Institute; Minato-ku, Tokyo, Japan).

## Results

### Patients’ clinicopathological characteristics

Table 1 shows patients’ characteristics. Pretreatment MRI revealed 34 RLNs in 16 patients. Table 2 shows the details of the anatomical location of these RLNs. All of them were lateral RLNs and there were no medial RLNs.

**Table.1:** Patients’ characteristics

Characteristics	Number of patients (%)
Age	
Median (range)	67 (48–81)
Gender	
Male	13 (81)
Female	3 (19)
T stage	
T4a	12 (75)
T4b	4 (25)
N stage	
N0	6 (38)
N1	1 (6)
N2b	9 (56)
Pretreatment diagnostic imaging	
MRI	16 (100)
PET-CT	9 (56)
Radiation thechnique	
3DCRT	9 (56)
IMRT	7 (44)

**Table.2:** Location of RLNs on pre-treatment MRI

Location on MRI	No. of RLNs at pretreatment MRI		No. of positive for metastatic RLNs	
	Ipsilateral	Contralateral	Ipsilateral	Contralateral
Occipital bone	3	1	3	–
Body of C1 and C1/2 disk	9	6	4	–
Body of C2 and C2/3 disk	6	8	–	–
Body of C3	–	1	–	–

**Fig. 1:**
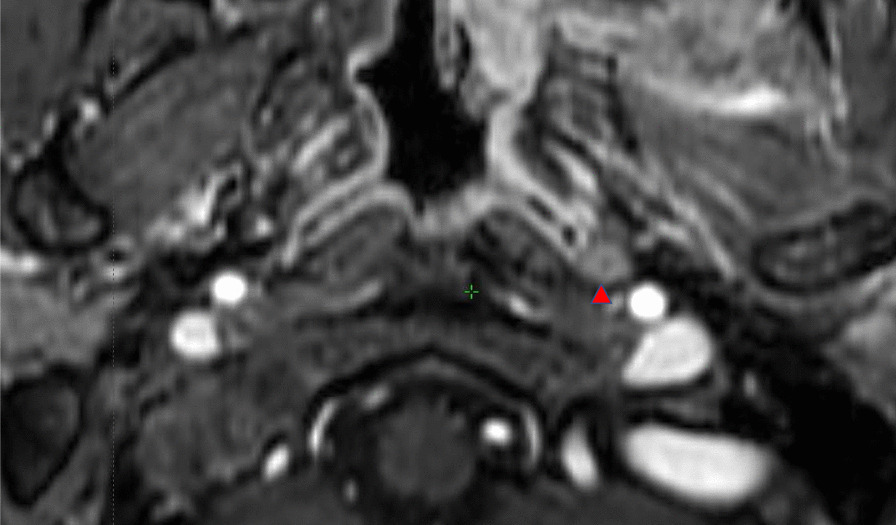
Metastatic RLN with findings of central necrosis. Arrowheads indicate RLNs with central necrosis

All patients had completed radiation therapy, and the total number of MRI f/u ranged from 1 to 7 (median, 2) over a median observation period of 38 months. The median intersection gap of the MRI axial image was 0.9 mm (range, 0.7–5.0). No cases had a local recurrence by the time of the final MRI f/u. Therapeutic response detected by the initial MRI after treatment was distributed as follows: CR, two LNs; PR, five LNs; and SD, 27 LNs. Subsequent MRI f/u showed no cases in which the evaluation changed from SD to PR or PD. On the basis of the therapeutic response detected by MRI f/u, seven RLNs were classified as metastatic lesions and 27 as non-metastatic lesions. The minimal axial diameters of the positive RLNs were greater than those of the negative RLNs (Table 3). Three RLNs showed central necrosis, all of which were positive for metastasis by image f/u (Fig. 1).

**Table.3:** Axial diameter of metastatic or non-metastatic RLNs

RLNs status	Minimal axial diameter	Maximal axial daiameter
Positive	5.9 ± 0.5	8.8 ± 1.1
Negative	3.4 ± 0.1	6.5 ± 0.3
p value	0.0001	0.06

### Radiologic criteria of metastatic retropharyngeal lymph nodes

**Fig. 2:**
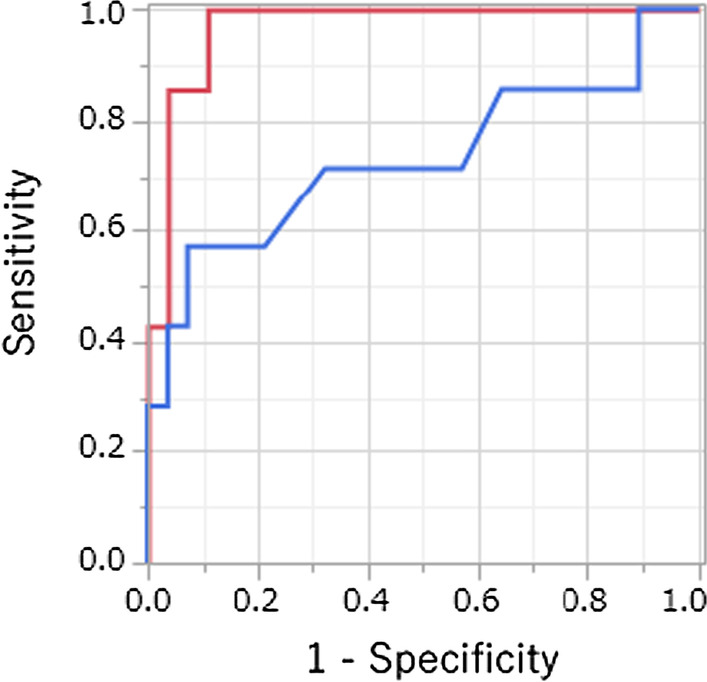
ROC curves of the maximal and minimal axial diameters. Red: minimal axial diameter; blue: maximal axial diameter. Metastatic RLNs could be diagnosed more accurately with the minimal axial diameter than with the maximal axial diameter (AUC 0.97 vs. 0.73, *p* = 0.05)

Figure 2 shows the ROC curves of the maximal and the minimal axial diameters. Metastatic RLNs tended to be diagnosed more accurately with the minimal axial diameter than with the maximal axial diameter (AUC 0.97 vs. 0.73, *p* = 0.06). Table 4 shows the sensitivity, specificity, and accuracy for several cut-off values per mm of the different size criteria. The most accurate radiologic criterion was a minimal axial diameter of 5 mm or larger. The sensitivity, specificity, and accuracy of this criterion were 85.7%, 96.3%, and 94.1%, respectively. In two RLNs, there was a discrepancy between the judgment of metastatic RLNs using this radiologic criterion and that based on the evaluation by MRI f/u. That is, the minimal axial diameter was 4.4 mm before treatment, but the therapeutic effect was CR. Conversely, the minimal axial diameter was 5.9 mm before treatment, but the therapeutic effect was SD.

**Table.4:** Sensitivity, specificity, and accuracy of several cut-offs per mm of different size criteria

Diameter (mm)	Sensitivity(%)	Specificity	Accuracy
	( n = 7)	( n = 28)	( n = 35)
Minimal axial diameter			
≥ 3 mm	100	22.2	38.2
≥ 4 mm	100	81.5	85.3
≥ 5 mm	85.7	96.3	94.1
≥ 6 mm	42.9	100	88.2
≥ 7 mm	14.3	100	82.3
Maxmal axial daiameter			
≥ 6 mm	71.4	44.4	50
≥ 7 mm	71.4	63	64.7
≥ 8 mm	57.1	81.5	76.5
≥ 9 mm	57.1	92.6	85.2
≥ 10 mm	57.1	92.6	85.2

## Discussion

There were few reports of RLNs in MSC before 2000 [1], but in recent years there have been reports of a high frequency of RLNs detected by MRI [14]. The inconsistent frequency of metastatic RLNs may be due to changes in diagnostic modalities and the diagnostic criteria [8]. In a previous study, a maximal axial diameter of 10 mm or longer was used as the diagnostic criterion for metastatic RLNs of head and neck cancer [18]. Recently, from the examination of RLNs in healthy subjects by MRI, a minimal axial diameter of 5 mm or longer was often used as the diagnostic criterion [19, 20]. However, it is controversial to extrapolate the MRI findings of healthy individuals to the diagnostic criteria of cancer patients. Regarding RLN for MSC, there are reports of diagnostic criteria of 5 mm or more for the minimal axial diameter and 8 mm or more for the major axial diameter, and the diagnostic criteria are not constant [14, 21]. Zhang et al. reported that the optimal diagnostic criterion was a minimal axial diameter of 6 mm or longer for metastatic RLNs in nasopharyngeal cancer on the basis of the treatment response by MRI before and after treatment [22]. Pathological findings in the RLN region are difficult to obtain due to the risk of adverse events such as vessel and nerve damage and dysphagia by dissection, and it is particularly useful to determine the diagnostic criteria for metastatic RLNs by imaging f/u [23]. Therefore, we revealed the optimal diagnostic criteria for RLNs for MSC from MRI f/u.

Judgment of metastatic RLNs by MRI f/u was determined according to RECIST ver. 1.1. Notably, in the case of continuous SD, the pretreatment image and the most changed image were compared to prevent an erroneous SD judgment. As a result, there were no cases of SD to PR or PD. On the basis of the therapeutic response, seven in 34 RLNs were classified as positive for metastasis. RLNs are classified as lateral and medial, and medial RLNs are usually judged to be metastatic by visual recognition, regardless of size [24]. There was no medial RLN, and thus there was no effect on the size in the radiologic criteria. Although not statistically significant, the maximal axial diameters of metastatic positive RLNs tended to be greater than those of negative RLNs, which reflected the findings of Zhang et al. Metastatic RLNs were more common at the occipital bone level than in previous studies [20], which may be because the primary site of this study included skull base invasion in many cases. The predictive value between anatomical location was not found (data not shown). All these metastatic RLNs were present on the ipsilateral side despite being advanced cases, and this result may indicate that prophylactic irradiation of the RLN region is sufficient on the ipsilateral side alone.

From the ROC curve analysis, metastatic RLNs tended to be diagnosed more accurately with the minimal axial diameter than with the maximal axial diameter. This was consistent with previous studies of RLNs in head and neck cancer [22, 24]. The most appropriate radiologic criterion was a minimal axial diameter of 5 mm or more, similar to the results reported by Zhang et al. in nasopharyngeal cancer. Thus, the diagnostic criterion revealed by this study was the same as those commonly used for head and neck cancer from the examination of RLNs in healthy subjects by MRI [19, 20]. Regarding the case with false positive in this criterion, the therapeutic response was judged to be SD. However, the f/u was performed only once, and caution is required in its interpretation. There were three RLNs with central necrosis, all of which were metastatic RLNs by MRI f/u and the specificity was 100%. The minimal axial diameter in one of three cases with central necrosis was 4.4 mm, and the addition of this finding to the diagnostic criterion by minimal diameter increased sensitivity slightly. However, the only findings of central necrosis of a small LN with a minimal axial diameter of approximately 5 mm were considered to have low sensitivity. That is, it was determined that the findings of central necrosis could be replaced by the criterion of minimal diameter, because only one case of central necrosis deviated from the criterion by minimal diameter.

This study has several limitations associated with its retrospective design. First, the modality and protocol of MRI performed before and after treatment were not standardized. Second, no pathological examination was performed, and positive metastasis was only identified by MRI f/u.

In this study, no RLN recurrence was observed, and the necessity of irradiation in the RLN region remains unknown. In recent years, there have been several reports of RLN recurrence (Table 5) [25–29], and in the future, the requirement for irradiation in the RLN region should be examined by evaluating the recurrence type using our radiologic criteria, taking into consideration any adverse events.

**Table.5:** Reports of recurrence of RLNs

	Total number	Number of RLN recurrences (%)	Stage	Pathology	Treatment	Radiologic criteria on metastatic RLN
Kimura et al. 1998	NR	2	T3N1M0, T3N0M0	SCC, ME	S + IART, S + AC	NR
Le et al. 2000	97	2 [2]	T3 or T4	NR	NR	NR
Umeda et al. 2005	22	1 [5]※	T2N0M0	SCC	S + IART	NR
Homma et al. 2014	98	2 [2]	T4N0M0	SCC	S + RT	NR
Jeon et al. 2017	71	1 [1]※	NR	NR	NR	NR

※contralateral side recurrence

SCC; squamous cell carcinoma, ME; mucoepidermoid cancer, S; surgery, IART; intraarterial chemotheraoy and radiation therapy

## Conclusion

In conclusion, the most appropriate radiologic criterion of metastatic RLNs in patients with MSC is a minimal axial diameter of 5 mm or more detected by MRI f/u.

## Data Availability

The data that support the findings of this study are available on request from the corresponding author.
